# Effects of five hindfoot arthrodeses on foot and ankle motion: Measurements in cadaver specimens

**DOI:** 10.1038/srep35493

**Published:** 2016-10-18

**Authors:** Kun Zhang, Yanxi Chen, Minfei Qiang, Yini Hao

**Affiliations:** 1 Department of Orthopedic Trauma, East Hospital, Tongji University School of Medicine, 150 Jimo Rd, Shanghai 200120, China

## Abstract

Single, double, and triple hindfoot arthrodeses are used to correct hindfoot deformities and relieve chronic pain. However, joint fusion may lead to dysfunction in adjacent articular surfaces. We compared range of motion in adjacent joints before and after arthrodesis to determine the effects of each procedure on joint motion. The theory of moment of couple, bending moment and balanced loading was applied to each of 16 fresh cadaver feet to induce dorsiflexion, plantarflexion, internal rotation, external rotation, inversion, and eversion. Range of motion was measured with a 3-axis coordinate measuring machine in a control foot and in feet after subtalar, talonavicular, calcaneocuboid, double, or triple arthrodesis. All arthrodeses restricted mainly internal-external rotation and inversion-eversion. The restriction in a double arthrodesis was more than that in a single arthrodesis, but that in a calcaneocuboid arthrodesis was relatively low. After triple arthrodeses, the restriction on dorsiflexion and plantarflexion movements was substantial, and internal-external rotation and inversion-eversion were almost lost. Considering that different arthrodesis procedures cause complex, three-dimensional hindfoot motion reductions, we recommend talonavicular or calcaneocuboid arthrodesis for patients with well-preserved functions of plantarflexion/dorsiflexion before operation, subtalar or calcaneocuboid arthrodesis for patients with well-preserved abduction/adduction, and talonavicular arthrodesis for patients with well-preserved eversion/inversion.

Correcting complex hindfoot deformities to relieve chronic pain from osteoarthritis or posttraumatic cartilage degeneration commonly requires a single, double, or a triple arthrodesis^1^. Compared with procedures involving osteotomy, the risk of morbidity in arthrodesis is lower during, as well as after, surgery. Arthrodeses usually cause less immobilization and are easier to perform^2^.

Functional evaluation of the biomechanics of hindfoot arthrodesis has identified surgical indications and techniques for different types of arthrodesis^3^,^4^,^5^,^6^,^7^. The high rate of partial failure of hindfoot motion after arthrodesis^6^,^8^,^9^,^10^,^11^ is related to foot pain and the loss of functional stability. Fortin^4^ reported that subtalar joint motion was reduced by 80% to 90% after an isolated fusion of the talonavicular joint and that motion of the calcaneocuboid joint was lost completely, leading to accelerated arthrosis of adjacent joints. In another study, after an isolated subtalar fusion, the range of motion (ROM) of the subtalar joint was reduced by 20%, that of the calcaneocuboid joint was reduced 44%, and that of the talonavicular joint, by 74%^1^.

Astion *et al*. found that simulated arthrodesis of the calcaneocuboid joint had little effect on the ROM of the subtalar joint, but it reduced the ROM of the talonavicular joint to 67%^10^. Similarly, Harper and Tisdel found that after simulated double arthrodesis, only 30% the motion of the subtalar joint was retained. In addition, plantar flexion of the ankle after an isolated fusion of the talonavicular joint was reduced by 10%^12^. Finally, transverse tarsal motion was diminished by 40%, dorsiflexion by 30%, and plantarflexion by 9% after an isolated subtalar arthrodesis^13^. However, the methodologies of the above investigations were different, which makes comparison difficult.

Unfortunately, accurately measuring the ROM in the foot and ankle is difficult after arthrodesis in the clinical setting. The past few years, several foot models have been published^14^, including cadaveric arthrodesis models^1^,^7^. These models have been used to assess dynamic foot motion to refine indications and assess the outcomes of surgical or conservative management, such as botulinum toxin treatment and serial casting^15^,^16^.

However, the amount of data on typical foot motion is limited, especially after various types of arthrodesis. To collect these data, we used a cadaver model of the non weight-bearing foot and ankle and measured the ROM of the various movements of the foot before and after subtalar, talonavicular, calcaneocuboid, double, and triple arthrodesis. These results can be used to determine the least-invasive and most biomechanically sound treatment for hindfoot pain and deformity.

## Results

Evaluable measurements were obtained in all planes of movement from all 16 specimens. Creep of the specimens after surgery was considered basically the same when the preservation and thawing conditions of specimens were well controlled. No loading condition was destructive. Two-way repeated-measures ANOVA showed a significant effect of both different arthrodesis (*F* = 1357.28, df = 3.48, *P* < 0.001, Greenhouse-Geisser corrected) and force loading (*F* = 2307.54, df = 3.96, *P *< 0.001, Greenhouse-Geisser corrected), as well as a significant interaction of different arthrodesis and force loading (*F *= 113.0, df* *= 17.41, *P *< 0.001, Greenhouse-Geisser corrected). In almost all cases, the arthrodesis significantly reduced ROM in all directions (Tables 1 through 5). In subtalar arthrodesis (Table 1), the decrease was especially great in inversion-eversion. Talonavicular arthrodesis (Table 2) significantly reduced ROM in all directions with various degrees, save for dorsiflexion (*P *= 0.27). The greatest reduction was observed in internal-external rotation motions. In calcaneocuboid arthrodesis (Table 3), the decrease was especially great in internal rotation and eversion motions. Double arthrodesis (Table 4) significantly reduced ROM in all directions. The greatest reduction was observed in inversion-eversion and internal-external rotation motions. Triple arthrodeses (Table 5) significantly reduced ROM in all directions. The ROM in inversion-eversion and internal-external rotation were almost completely lost.

## Discussion

The optimal treatment of hindfoot disease is often a matter of debate. For example, in treating a traumatic deformity of the calcaneus, opinions differ on whether an arthrodesis of the cuboid and calcaneus should be added to a subtalar arthrodesis^9^. Likewise, lengthening the lateral column of the foot corrects a flatfoot deformity; however, whether the cuboid and calcaneus should be fused is not clear^3^,^17^,^18^. In a severe flatfoot deformity, triple arthrodesis can produce a stable plantigrade foot^7^.

Although techniques can preserve more of the normal hindfoot mechanics^19^, choosing between a single or double arthrodesis for partial hindfoot mobility and a triple fusion for full hindfoot immobilization is not straightforward. Using a model that approximates the *in-vivo* physiological state of the foot and ankle, we evaluated different arthrodeses of the hindfoot joint to provide a more reliable basis for making these decisions.

In other cadaveric studies of ankle biomechanics, fresh specimens were often fixed on an experimental frame or to a test bench with a Kirschner wire^10^,^20^,^21^,^22^. However, even if a person stands still, the foot joints slide and rotate slightly to balance the body’s weight^23^. Fixing the foot in a test bench can immobilize the joint before the external force is applied, generating an unnatural resistance that might reduce the accuracy of the experiment. Therefore, the ideal biomechanical model should be the least restrictive to best approximate normal anatomy and physiology and to provide the most accurate measurements.

In this study, we applied the “least-restrictive” principle when testing the cadaver specimens to better approximate the normal moment of coupling, bending moment, and load-balancing. Our experimental setup enabled the hind foot to move more-or-less normally in all three planes. However, in the loading process, the moment of force from all directions had to be adjusted to ensure that it would not produce any obvious additional movement in the forefoot. For instance, when the forefoot base plane dorsiflexes and plantarflexes relative to the horizontal plane of the test bench, the moments of force from all directions were adjusted to avoid the internal-external rotation and inversion-eversion of forefoot relative to the vertical and sagittal axis of the tibial shaft.

Because the experiment was focused on the hindfoot joints, we chose to apply the forces to the forefoot. Thus, the force moved the forefoot relative to the hindfoot, the forefoot relative to the tibial shaft, and the hindfoot relative to the tibial shaft. Therefore, it was easier to measure the general movement of the forefoot relative to the tibia to compare the maximum normal ROM before and after each hindfoot arthrodeses. When the forefoot was loaded to the limit of movement in one direction, the angle of the forefoot relative to the tibia was measured with a special three-axis protractor.

Few studies have considered the impact on general foot movement after mid-hindfoot arthrodeses. The particularity and complexity of foot movement have given rise to different kinematic terms^24^, which to some extent reflects the contrast between basic and clinical research. Lee reported that the axis of forefoot motion differs from that of the rearfoot motion; thus, the general degree of foot movement for each axis cannot be described with a single set of terms^25^. Nevertheless, the purpose of our study was to investigate the impact of different arthrodeses on the foot movement, so we described the movement of the forefoot relative to the tibia. Although we measured only six simple movements, every motion of the foot is a combination of these six movements.

The human foot is often modeled as a rigid body in gait analysis. However, this representation is oversimplified, given that the foot is a highly complex structure of 29 bones, 33 joints, and more than 100 muscles, tendons, and ligaments^26^. In reality, the various structures of the foot can move relative to each other. Motion of the so-called triple joint complex (the subtalar, talonavicular, and calcaneocuboid joints) is necessary for the foot to accommodate variations in ground surface and leg rotation^10^. For example, the subtalar joint, one of the most complex weight-bearing joints, converts the rotational forces of the leg into calcaneus and dictates the movements of the mid-tarsal joints and the forefoot.

Minor biomechanical or anatomical details can have considerable clinical importance^6^,^16^. The relative motion between the hindfoot and the forefoot occurs at the transverse tarsal joint (or mid-tarsal joint or Chopart’s articulation), consisting of the talonavicular and calcaneocuboid joints. The talonavicular joint has the greatest ROM of the triple joint complex^4^.

According to the results, the variation of ROM of foot with different force loading procedures was influenced by different arthrodesis. We found that the effect of subtalar, talonavicular, calcaneocuboid, and double (calcaneocuboid and talonavicular) arthrodeses on dorsiflexion and plantarflexion in non-weight-bearing foot specimens restricted internal-external rotation. Subtalar arthrodesis restricted inversion-eversion ROM by 84% and 88%, respectively, and talonavicular arthrodesis restricted internal-external rotation by 50% and 62%, respectively. The restriction in a double arthrodesis was more than that in a single joint arthrodesis, but that in a calcaneocuboid arthrodesis was relatively low. After triple arthrodeses, the restriction on dorsiflexion and plantarflexion movements was substantial, and internal-external rotation and inversion-eversion were almost lost. The restriction rate was 33% in dorsiflexion, 18% in plantarflexion, 93% in adduction, 95% in abduction, 94% in inversion, and 90% in eversion.

One limitation of this study was the small number of specimens and age groups represented. Another was that we measured movement in only six directions because it was too difficult to simulate the complex motions of the foot and to reach a steady state where reliable measurements could be taken.

## Conclusion

In sum, creating a cadaver model that approximates natural human physiological conditions is difficult. Therefore, we did not try to measure the ROM of the foot and ankle with a high degree of accuracy but rather compared these measurements taken before and after the arthrodeses. Clinicians could use these results to determine the least-invasive and most biomechanically sound treatment for hindfoot-related diseases. Creating a biomechanical model that approximates normal human physiology and biomechanics that can simulate the dynamic forces on the foot is our goal.

## Materials and Methods

### Ethics Statement

The experiments were carried out in accordance with the guidelines of the Declaration of Helsinki and all experimental protocols were approved by the Regional Ethical Committee (Tongji Hospital Ethics Committee, Ethics number LL (H)-08-02). The subjects gave informed consent and patient anonymity has been preserved. All consent was written in nature, and where deceased, written consent was obtained from the next of kin of the deceased.

### Cadaver models

We studied 16 fresh-frozen cadaver feet that had been amputated above the ankle from 10 male and 6 female patient cadavers (5 left feet and 11 right feet). The tibia and fibula were amputated at the junction of the middle and distal thirds. The skin, subcutaneous tissues, and muscle were dissected from the most proximal portion. The interosseus and ligaments of the foot and ankle were kept intact. The average age at the time of death was 37 years (range, 18 to 58 years). There was no macroscopic or radiographic evidence of injury, surgeries, osteoarthritis, or severe deformities. Specimens were stored at −80 °C and thawed to room temperature at the beginning of the experiment. The study protocol was approved by the institutional review board of Tongji Hospital Ethics Committee. (Ethics number LL (H)-08-02).

### Position of the specimens during testing

**Figure 1:**
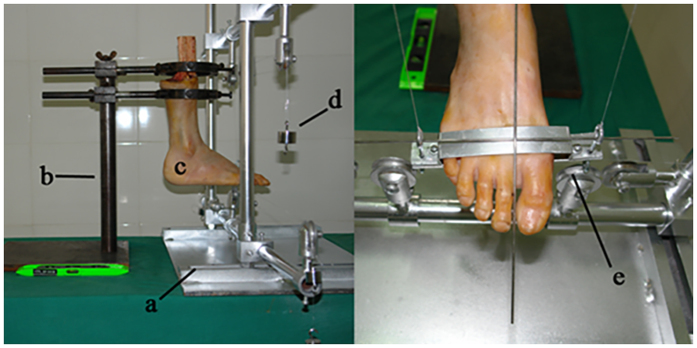
Specimens in the neutral position. Biomechanical loading bracket: (**a**); Specimen holder: (**b**); Specimen foot: (**c**); Weight: (**d**); Forefoot bracket: (**e**).

Each specimen was immobilized with a specimen holder consisting of a pedestal, a vertical bar, and two steel rims with handles fixed to the vertical bar, along which the foot could be rotated and moved vertically (see Fig. 1). The position of the tibia and fibula could be changed by adjusting the bolts and handles. The forefoot was supported by two aluminum plates to determine the degree of plantarflexion in the forefoot. When the forefoot was fixed, the frontal axis of the bracket must be perpendicular to the sagittal axis of the foot.

We defined the neutral position in 3 axes: neutral dorsiflexion-plantarflexion was defined as 0° between the long axis of the tibia-fibula and a line perpendicular to the plantar aspect of the foot projected onto the sagittal plane of the tibia-fibula. Neutral inversion-eversion was defined as 0° between the long axis of the tibia-fibula and a line perpendicular to the plantar aspect of the foot projected onto the frontal plane of the tibia-fibula. Neutral internal-external rotation was defined as 0° between a line perpendicular to the frontal plane of the tibia-fibula and the long axis of the second metatarsal, projected onto the transverse plane of the tibia-fibula^27^. The neutral position was treated as the initial position of a foot with full ROM in all directions.

### Force loading and calculations

**Figure 2:**
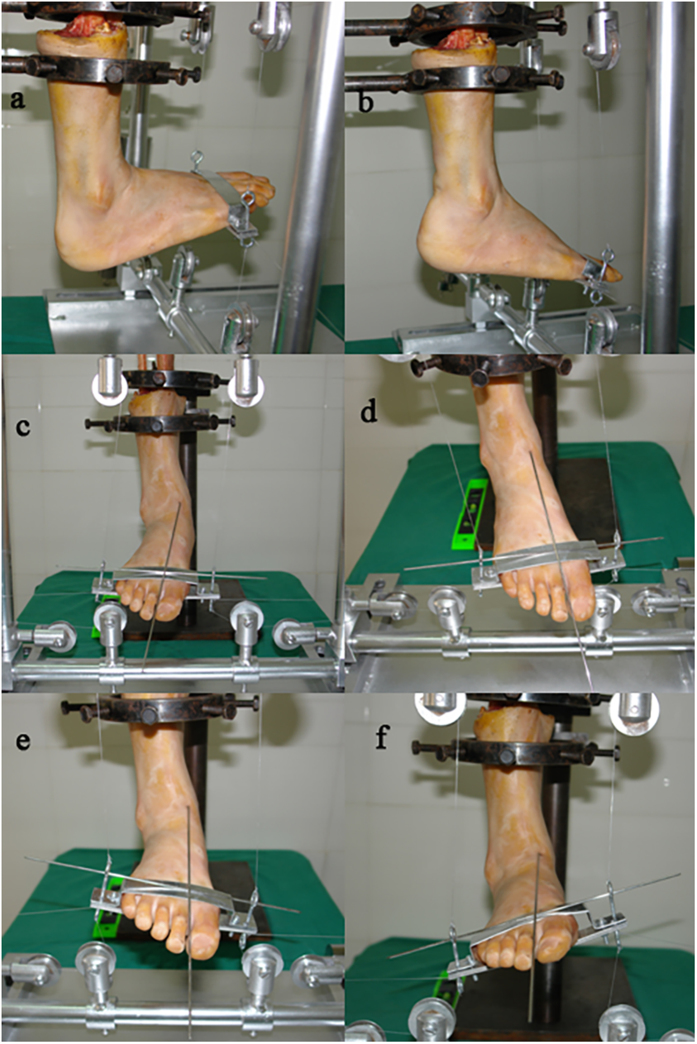
Specimen positions during testing. Dorsiflexion: (**a**); Plantarflexion: (**b**); Abduction: (**c**); Adduction: (**d**); Eversion: (**e**); Inversion: (**f**).

Because it is difficult to reach a steady-state when simulating the load of the foot, we applied a force in six general motions: dorsiflexion-plantarflexion, internal-external rotation, and inversion-eversion. Load theory: Ι. The moment of couple from opposite direction was superimposed on the specimen foot to reach a steady state under the balance load; ΙΙ. The bending moment was superimposed up, down, left and right to the forefoot on the basis of balance load to achieve the steady state of the extreme sports with the forefoot extremely dorsiflexed-plantarflexed and internally-externally rotated relative to the coronal and vertical axis of the tibia; ΙΙΙ. The size of either moment of couple was changed to rotate the forefoot to achieve the steady state of the extreme sports when it extremely inverted-everted relative to the sagittal axis of tibial shaft (see Fig. 2).

Force was applied in progressive smaller increments until there was no visible movement for 10 seconds. This position was considered to be the maximum ROM in that movement. The force was then stopped, and the load-deformation curve was drawn. To prevent the force from damaging the internal structure of the foot, we repeated the above procedure. When the load-deformation curve reached a plateau, the limit load was considered to be effective.

We measured the ROM of the foot with a custom-made device (see Supplementary Fig. S1). The device held the foot on a plastic plate that could be rotated in all three planes. The ROM in all three planes was measured with a protractor.

### Arthrodesis procedures

All surgeries were performed by Chen with 14 years of experience in foot surgery. The damage to the soft tissues in the mid-hindfoot should be minimized during arthrodesis. We used standard clinical techniques and hardware (Kanghui LTD, Changzhou, China) to perform each arthrodesis^7^, with the exception that the articular surfaces were left intact. Each arthrodesis consisted of the following procedures. First, a subtalar arthrodesis was performed, in which the calcaneus was everted relative to the talus by 5° to 10° using two half-threaded cancellous screws 6.5 mm in diameter and 75 mm long and one half-threaded cancellous screw 6.5 mm in diameter and 70 mm long inserted from the calcaneus, across the subtalar joint and into the talus.

Second, we performed a talonavicular arthrodesis by dissecting the deep fascia and exposing the tibial anterior tendon and the extensor hallucis longus without opening the joint capsule. We placed three, fully threaded cancellous screws from the navicular bone across the talonavicular joint into the talus.

**Figure 3:**
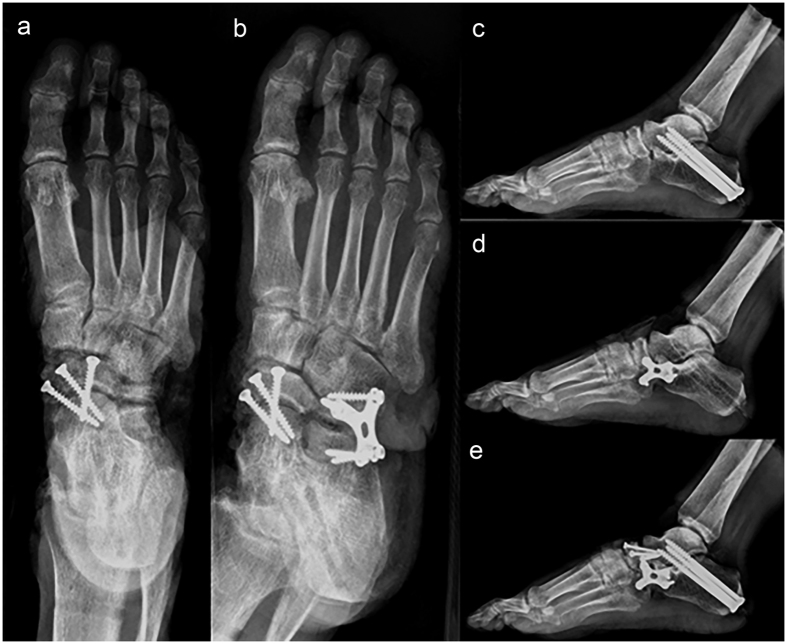
Standard clinical techniques and hardware used in all arthrodesis. Talonavicular: (**a**); calcaneocuboid + talonavicular: (**b**); subtalar: (**c**); calcaneocuboid: (**d**); triple arthrodesis: (**e**).

Third, in a calcaneocuboid arthrodesis, we dissected the deep fascia, retracted the peroneus brevis posterolaterally, and separated the belly of the extensor digitorum brevis by blunt dissection and retracted it superiorly without opening the joint capsule. With the foot in a neutral position, the calcaneocuboid joint was fixed with a butterfly-shaped titanium plate with fully threaded cancellous screws. Double (calcaneocuboid and talonavicular) and triple (calcaneocuboid, talonavicular, and subtalar) arthrodeses were performed by simply combining the corresponding procedures described above (Fig. 3).

For all procedures, postoperative radiographs were taken to confirm that the joint was accurately aligned and the screws were well positioned and did not protrude into the joint space. In accordance with the preoperative design, the subtalar arthrodesis was performed first, followed by talonavicular arthrodesis, calcaneocuboid arthrodesis, calcaneocuboid plus talonavicular arthrodesis, and then triple arthrodesis. The reliability of the applied force and measuring techniques was investigated in a specimen with each type of arthrodesis as described previously. Measurements in each loading direction were repeated five times by Chen, and the reliability was tested using intraclass correlation coefficient (ICC). The final ICC was 0.86–0.97 (95% CI, 0.74–0.99), indicating a good reliability.

### Statistical methods

The Kolmogorov-Smirnov test was used to test for normality of the distribution. Ranges of motion before and after each arthrodeses were assessed with Bonferroni-corrected paired t-tests after two-way repeated-measures ANOVA. Only those *P* < 0.05/30 = 0.0017 was considered significant. The data were analyzed with SPSS 18.0 for Windows (SPSS Inc., Chicago, IL, USA).

The percentage of reduction in movement after the arthrodesis was calculated as follow:


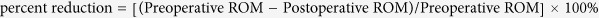


## Additional Information

**How to cite this article**: Zhang, K. *et al*. Effects of five hindfoot arthrodeses on foot and ankle motion: Measurements in cadaver specimens. *Sci. Rep.* **6**, 35493; doi: 10.1038/srep35493 (2016).

## Supplementary Material

Supplementary Dataset 1

Supplementary Dataset 2

## Figures and Tables

**Table 1:** Changes in Range of Foot Motions after Subtalar Arthrodesis (n = 16).

Position	Maximum range of motion, degrees		*P*	Restriction rate (%)	
	Preoperative angle, mean (SD)	Postoperative angle, mean (SD)			
Dorsiflexion	34.62 (2.73)	25.90 (2.63)	<0.001	25.11 (3.89)	
Plantarflexion	45.18 (3.12)	40.17 (3.21)	<0.001	11.17 (2.03)	
Abduction	21.85 (2.95)	12.15 (2.03)	<0.001	44.95 (4.23)	
Adduction	23.80 (3.10)	16.05 (2.86)	<0.001	32.54 (3.25)	
Eversion	20.32 (2.68)	2.39 (0.62)	<0.001	88.14 (7.55)	
Inversion	37.55 (3.03)	6.25 (1.73)	<0.001	83.55 (6.28)	
Mean square	3959.39				

**Table 2:** Changes in Range of Foot Motions after Talonavicular Arthrodesis (n = 16).

Position	Maximum range of motion, degrees		*P*	Restriction rate (%)	
	Preoperative angle, mean (SD)	Postoperative angle, mean (SD)			
Dorsiflexion	34.62 (2.73)	33.92 (2.73)	0.27	NS	
Plantarflexion	45.18 (3.12)	42.15 (3.32)	<0.001	6.67 (1.85)	
Abduction	21.85 (2.95)	8.27 (1.53)	<0.001	62.11 (8.32)	
Adduction	23.80 (3.10)	12.05 (2.89)	<0.001	49.50 (6.33)	
Eversion	20.32 (2.68)	14.79 (1.97)	<0.001	27.15 (4.52)	
Inversion	37.55 (3.03)	24.32 (2.36)	<0.001	34.90 (5.95)	
Mean square	4216.29				

**Table 3:** Changes in Range of Foot Motions after Calcaneocuboid Arthrodesis (n = 16).

Position	Maximum range of motion, degrees		*P*	Restriction rate (%)
	Preoperative angle, mean (SD)	Postoperative angle, mean (SD)		
Dorsiflexion	34.62 (2.73)	33.17 (2.64)	0.02	4.04 (2.29)
Plantarflexion	45.18 (3.12)	43.90 (3.14)	<0.001	2.76 (2.57)
Abduction	21.85 (2.95)	13.38 (2.39)	<0.001	37.50 (4.36)
Adduction	23.80 (3.10)	18.95 (3.62)	<0.001	20.32 (4.68)
Eversion	20.32 (2.68)	9.63 (2.55)	<0.001	52.11 (6.42)
Inversion	37.55 (3.03)	29.02 (3.85)	<0.001	22.64 (4.07)
Mean square	4205.67			

**Table 4:** Changes in Range of Foot Motions after Double Arthrodesis (n = 16).

Position	Maximum range of motion, degrees		*P*	Restriction rate (%)
	Preoperative angle, mean (SD)	Postoperative angle, mean (SD)		
Dorsiflexion	34.62 (2.73)	28.36 (3.32)	<0.001	18.01 (3.77)
Plantarflexion	45.18 (3.12)	42.55 (4.02)	<0.001	5.80 (2.45)
Abduction	21.85 (2.95)	5.36 (1.96)	<0.001	75.04 (8.99)
Adduction	23.80 (3.10)	4.55 (2.03)	<0.001	80.00 (9.27)
Eversion	20.32 (2.68)	6.35 (2.83)	<0.001	69.02 (7.88)
Inversion	37.55 (3.03)	12.57 (3.98)	<0.001	66.45 (7.32)
Mean square	4912.78			

**Table 5:** Changes in Range of Foot Motions after Triple Arthrodesis (n = 16).

Position	Maximum range of motion, degrees		*P*	Restriction rate (%)	
	Preoperative angle, mean (SD)	Postoperative angle, mean (SD)			
Dorsiflexion	34.62 (2.73)	23.08 (3.02)	<0.001	33.25 (4.78)	
Plantarflexion	45.18 (3.12)	37.45 (4.10)	<0.001	17.87 (2.66)	
Abduction	21.85 (2.95)	1.15 (0.34)	<0.001	94.75 (4.32)	
Adduction	23.80 (3.10)	1.75 (0.68)	<0.001	92.73 (6.01)	
Eversion	20.32 (2.68)	2.01 (1.03)	<0.001	90.03 (7.98)	
Inversion	37.55 (3.03)	2.31 (1.14)	<0.001	94.01 (5.35)	
Mean square	4675.21				
