# Supplementary material for: Effects of five hindfoot arthrodeses on foot and ankle motion: Measurements in cadaver specimens
Source: Sci Rep. 2016 Oct 18;6:35493. doi: 10.1038/srep35493 (PMC5067490; doi:10.1038/srep35493)
Supplement: Supplementary Dataset 2 [file srep35493-s2.doc]

**Manuscript Title:**

Effects of five hindfoot arthrodeses on foot and ankle motion: Measurements in cadaver specimens

**Authors' names in the order:**

Kun Zhang, MD

Yanxi Chen^*^, Phd, MD

Minfei Qiang, MD

Yini Hao, MD

**Supplementary Figure S1**

**
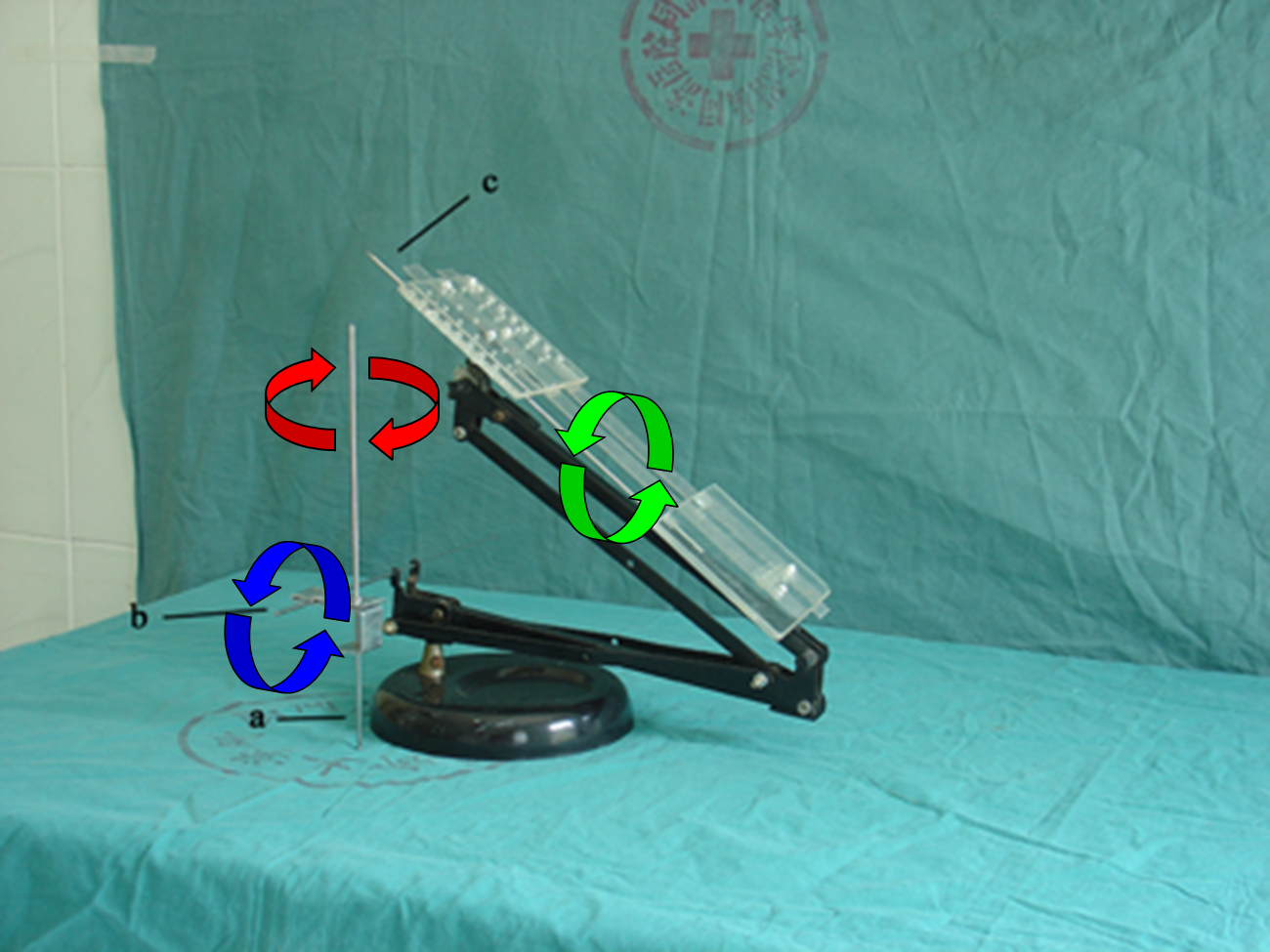
**

**Figure Legends**

**Supplementary Figure S1.** A device for measuring foot position in all three dimensions. The angles of abduction-adduction angle (red arrows), dorsiflexion-plantarflexion (blue arrows), and inversion-eversion (green arrows) of each foot were measured with a protractor around rotation of the a) vertical axis, b) transverse axis, and c) sagittal axis.
